# Computed Tomographic Findings and Clinical Manifestations of Acute Severe Hypersensitivity to a Non-Ionic Iodinated Contrast Agent in a Castrated Male Dog: A Case Report

**DOI:** 10.3390/ani16172708

**Published:** 2026-09-01

**Authors:** Minsoo Chung, Jungmin Kwak, Kidong Eom, Jaehwan Kim

**Affiliations:** 1 Department of Veterinary Medical Imaging, College of Veterinary Medicine, Konkuk University, Seoul 05029, Republic of Korea; ms3871@konkuk.ac.kr (M.C.); eomkd@konkuk.ac.kr (K.E.); 2 Human & Animal Medical Center, Yongin 16843, Republic of Korea; chocojae@naver.com

**Keywords:** anaphylactic shock, hypersensitivity, non-ionic contrast media, computed tomography, canine, vascular collapse, cardiopulmonary resuscitation

## Abstract

Severe adverse reactions induced by non-ionic contrast media remain an uncommon complication in veterinary medicine. This report describes the clinical manifestations and sequential computed tomography alterations indicative of acute shock captured during 2 min and 4 min delayed phases in a 10-year-old, castrated male Maltese dog. The patient, previously diagnosed with a cutaneous mast cell tumor on the lip exhibiting incomplete histopathological margins, underwent CT for metastatic evaluation. The patient had no clinical evidence of systemic disease, and no anesthetic adverse events were observed during past patellar luxation surgery. However, the 2 min delayed CT scan revealed suspected hepatic congestion, vascular collapse, marked esophageal wall edema, and intense intestinal mucosal contrast enhancement. Subsequently, the 4 min delayed CT scan demonstrated diffuse contrast medium stagnation within the cardiac chambers and renal parenchyma, accompanied by the absence of the renal excretory phase. During anesthetic recovery, severe hypotension and respiratory failure emerged, suggesting the development of hypersensitivity. Following immediate intervention with cardiopulmonary resuscitation, epinephrine, and dexamethasone, the patient temporarily exhibited facial edema, hematochezia, diarrhea, and transient ataxia. However, dobutamine administration successfully restored systemic blood pressure and tissue perfusion, returning the patient to a normal clinical status. Consequently, this case highlights potential pathophysiological mechanisms, key CT findings, and emergency management considerations for contrast-induced hypersensitivity. Notably, sequential CT imaging documented evolving shock-related changes before the onset of overt clinical deterioration, suggesting that these findings may represent early imaging indicators of contrast-induced hypersensitivity.

## Simple Summary

This case report describes a rare and severe hypersensitivity reaction in a 10-year-old Maltese dog associated with the administration of a non-ionic iodinated contrast medium commonly used during computed tomography scans. The dog, which was undergoing a computed tomography scan to check for cancer metastasis after a previous tumor removal, unexpectedly developed severe hypotension and respiratory failure. Real-time body scans captured during the emergency revealed significant internal changes, including a collapse of major blood vessels, severe swelling of the esophagus, and a stoppage of blood flow through the heart and kidneys. Following immediate life-saving treatments, including chest compressions, emergency medications, and a blood pressure support agent, the dog successfully made a full clinical recovery. As advanced imaging becomes more frequent in veterinary clinics, this study highlights the vital necessity for veterinary professionals to recognize these rare allergic complications instantly and to be fully prepared with rapid, life-saving rescue protocols. Ultimately, this report contributes to improving pet safety and enhancing the quality of emergency care in veterinary medicine.

## 1. Introduction

In veterinary medicine, contrast media have become indispensable for diagnostic imaging evaluations, including oncological staging, vascular diagnostics, interventional procedures, and radiation therapy planning, with their clinical utilization continuously rising. In particular, iodinated contrast agents, which facilitate lesion identification in radiography, fluoroscopy, and computed tomography (CT) via radiopacity, represent one of the most routinely employed contrast media in veterinary practice. In both human and veterinary medicine, the shift toward non-ionic iodinated contrast agents has progressively accelerated, as ionic agents carry a significantly higher risk of adverse reactions primarily driven by their high osmolality [1,2,3,4]. Adverse reactions to contrast media are divided into acute (within 1 h) and delayed (1 h to 7 days) reactions, and are further classified as mild, moderate, or severe based on clinical severity [3,5,6]. Generally, mild reactions (Grade I) include nausea, vomiting, flushing, pruritus, urticaria, and headache; most are self-limiting and resolve without specific medical intervention [5,6,7]. Moderate reactions (Grade II) involve severe vomiting, generalized urticaria, bronchospasm, facial or laryngeal edema, and hypotension, requiring appropriate medical treatment [3,6,7]. Severe reactions (Grade III~V) manifest as hypotensive shock, respiratory arrest, cardiac arrest, and seizures [3,5,6,7]. These are life-threatening emergencies that necessitate immediate resuscitative intervention. Although the incidence of severe adverse reactions associated with non-ionic iodinated contrast agents is remarkably lower than that of ionic agents, the risk is not entirely eliminated [2,8,9,10]. In veterinary medicine, several cases of severe adverse reactions induced by non-ionic iodinated contrast media have been documented [2,9,10].

This case report describes a canine patient that underwent general anesthesia for a computed tomography (CT) evaluation using a non-ionic iodinated contrast medium, where a progressive sequence of intra-procedural imaging features indicative of hemodynamic shock led to cardiopulmonary arrest and subsequent resuscitation. These imaging features demonstrate real-time CT changes that preceded clinical deterioration and may represent early imaging indicators of contrast-induced hypersensitivity. These findings highlight the potential value of veterinary radiologists in recognizing early imaging changes associated with clinical deterioration.

## 2. Case Description

### 2.1. Signalment, History, and Clinical Findings

A 10-year-old, 3.4 kg, castrated male Maltese dog was presented for a computed tomography (CT) evaluation. Two weeks prior to presentation, the patient had undergone surgical excision of a nodule on the left mandibular lip at a local referral hospital, which was histopathologically diagnosed as a low-grade mast cell tumor with incomplete surgical margins. On physical examination at the time of presentation, the regional lymph nodes were palpably normal. Fine-needle aspiration was not performed before CT, as the owner preferred FNA only if CT findings raised suspicion for lymph node metastasis. A contrast-enhanced whole-body CT imaging was scheduled for clinical staging to evaluate potential regional or distant metastasis.

Prior to the scan, the patient exhibited no clinical evidence of systemic disease based on physical examination, medical history, and routine pre-anesthetic laboratory investigations, including complete blood count and serum biochemistry, which revealed no clinically significant abnormalities. The patient was clinically stable with no remarkable cardiovascular history or known hypersensitivity to contrast agents. Prior to anesthesia, the dog had a stable spontaneous respiratory rate of 20 breaths/min. Additionally, there was no history of anesthetic complications during previous bilateral hindlimb surgeries for patellar luxation. Before the pre-contrast CT acquisition, following anesthesia induction and prior to contrast administration, the heart rate was 80–100 beats/min, systolic and diastolic arterial blood pressures were 110–120 mmHg and 70 mmHg, respectively, oxygen saturation (SpO_2_) was 98–100%, and end-tidal carbon dioxide (ETCO_2_) was 35 mmHg, all of which were within normal reference intervals. No remarkable findings were observed on pre-anesthetic radiography and ultrasonography. As a premedication, midazolam (0.2 mg/kg) was administered intravenously, and this represented the actual administered dose. Anesthesia was induced with an intravenous bolus of propofol (4 mg/kg), which also represented the actual administered dose, to facilitate endotracheal intubation, and subsequently maintained with isoflurane in 100% oxygen under controlled ventilation. No intravenous fluid therapy was administered before or during the CT examination. No complications arose during either procedure.

### 2.2. Imaging, Diagnosis and Outcome

**Figure 1:**
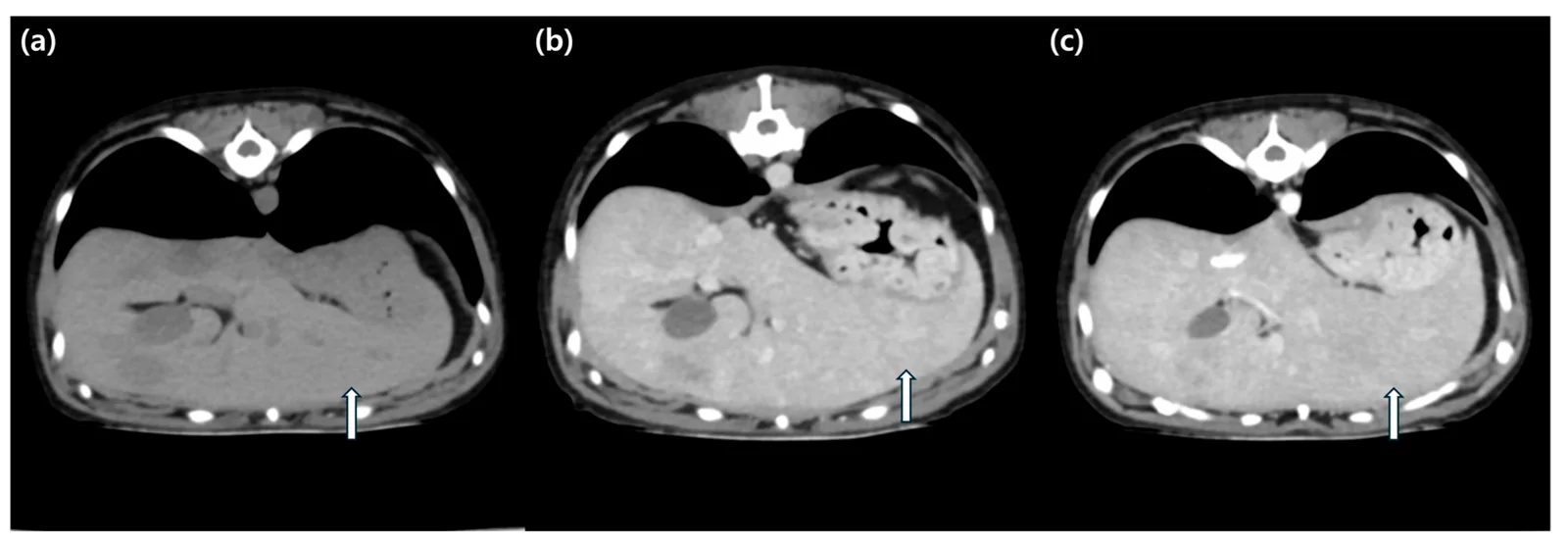
Hepatic contrast enhancement patterns at the level of the esophagogastric junction. (**a**) Pre-contrast image, showing homogeneous non-enhancing hepatic parenchyma (white arrow). (**b**) Two-minute delayed phase, where generalized heterogeneous contrast enhancement of the hepatic parenchyma (white arrow) is observed. (**c**) Four-minute delayed phase, demonstrating that the heterogeneous hepatic parenchymal enhancement (white arrow) remains similarly apparent.

**Figure 2:**
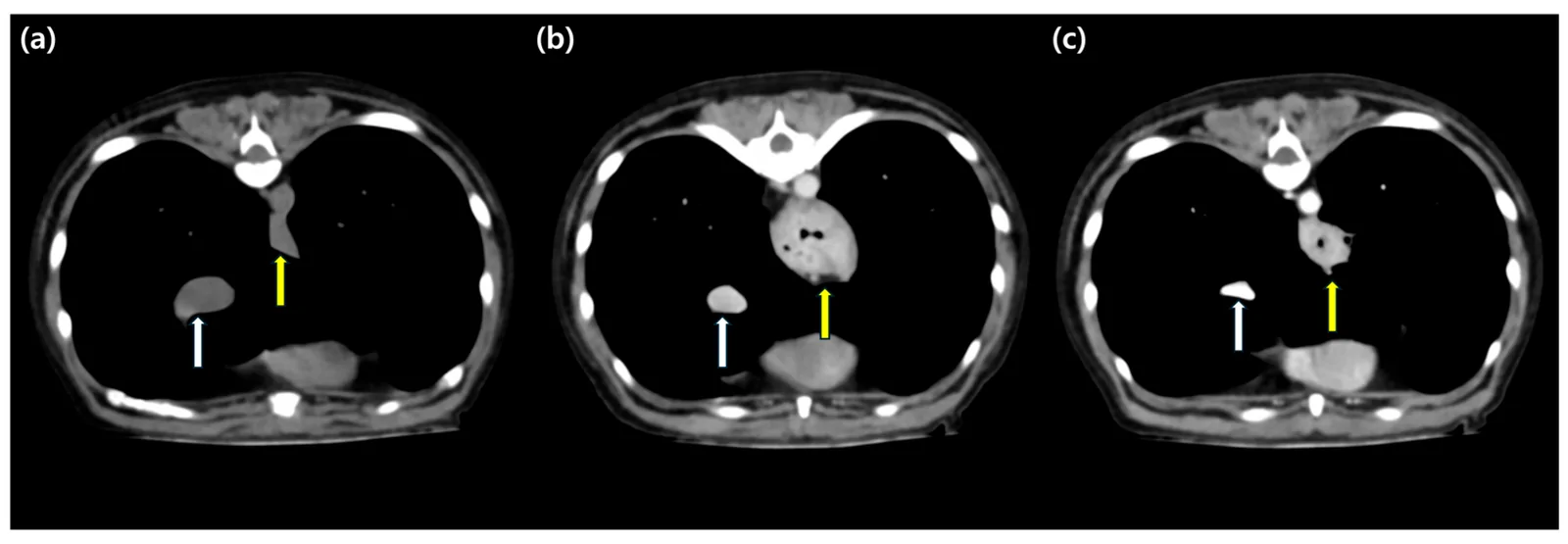
Morphological alterations of the esophagus (yellow arrows) at the level cranial to the esophagogastric junction and the caudal vena cava (CVC) (white arrows) at the level cranial to the diaphragm. (**a**) Pre-contrast image. A collapsed esophagus and a CVC maintaining an oval shape are observed. (**b**) Two-minute delayed phase. Compared to the pre-contrast image, severe thickening of the esophageal wall and mucosal layer is observed. Additionally, a drastic reduction in the CVC diameter is noticeable. (**c**) Four-minute delayed phase. Compared to the two-minute delayed phase, the esophageal wall thickening has decreased, but the CVC diameter exhibits a progressive reduction, demonstrating a slit-like appearance. Furthermore, the internal lumen of the CVC is hyperattenuated, which is a finding presumed to indicate contrast medium stagnation.

**Figure 3:**
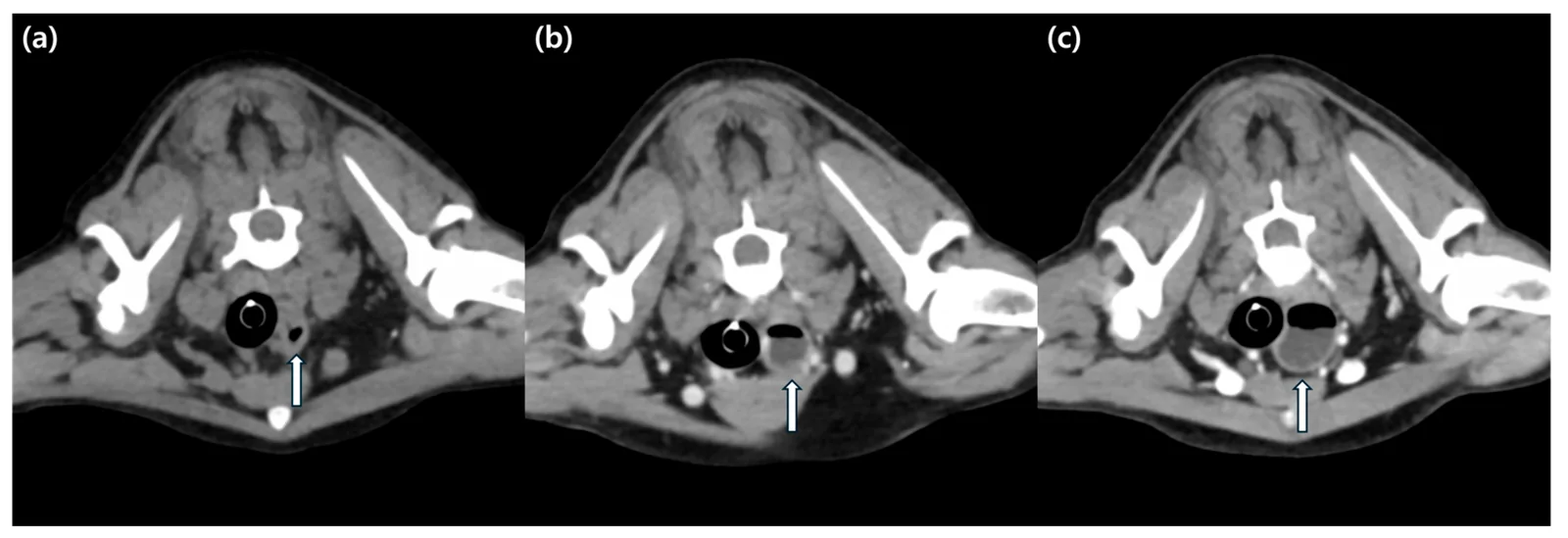
Morphological alterations of the esophagus (white arrows) at the level of the thoracic inlet. (**a**) Pre-contrast image. A lumen of the esophagus containing a small amount of air is observed. (**b**) Two-minute delayed phase. Dilation of the esophagus is observed, accompanied by the retention of fluid and air within the esophageal lumen. (**c**) Four-minute delayed phase. Compared to the two-minute delayed phase, the amount of retained fluid and air has increased.

**Figure 4:**
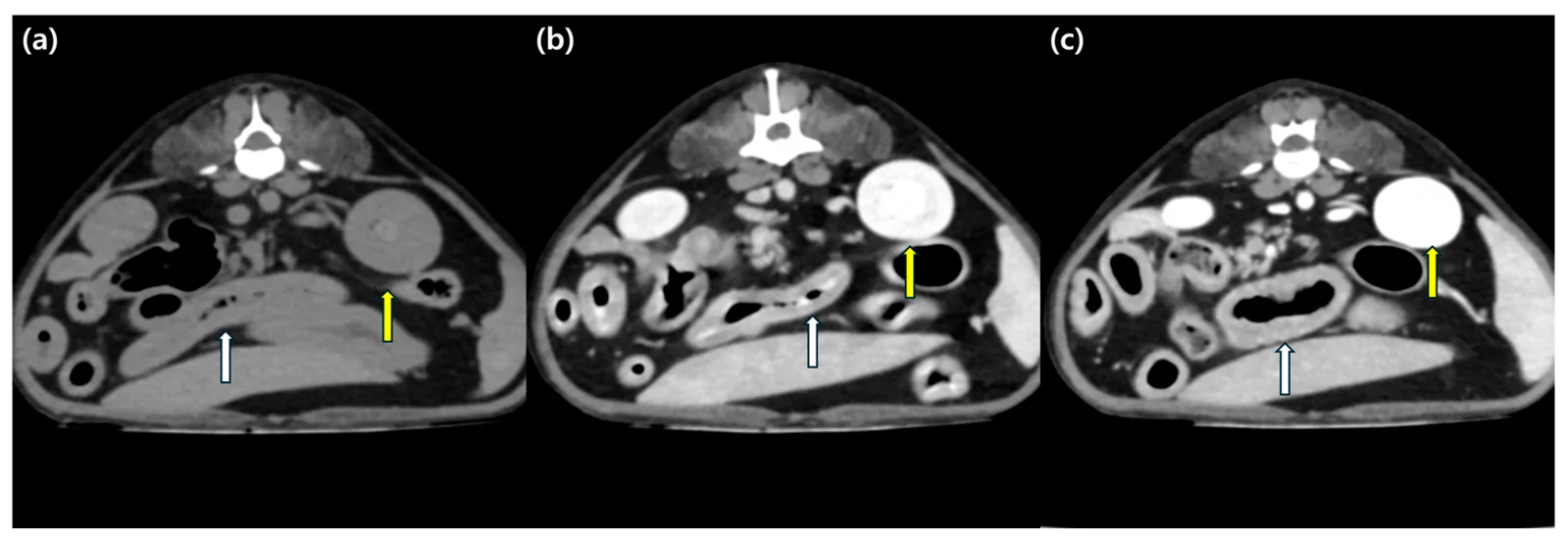
Contrast enhancement alterations of the small intestine (white arrows) and left kidney (yellow arrows) at the same level. (**a**) Pre-contrast image. (**b**) Two-minute delayed phase. Compared to the pre-contrast image, intense contrast enhancement of the small intestinal mucosal layer is observed. The left kidney exhibits strong contrast enhancement. (**c**) Four-minute delayed phase. Compared to the two-minute delayed phase, the intense enhancement of the small intestinal mucosal layer has resolved, while corrugation and luminal dilation due to gas are observed. The contrast enhancement of the left kidney appears even more pronounced.

For the CT examination, a non-ionic contrast medium, Iohexol (Omnipaque; 300 mg I/mL; GE Healthcare, South Plainfield, NJ, USA), was prepared at a dose of 2 mL/kg. The scan (Brilliance CT 16-slice; Philips, Amsterdam, The Netherlands) was conducted with the patient in sternal recumbency using the following parameters: 120 kVp, 150 mAs, 512 × 512 matrix, 1 mm slice thickness, 0.75 s rotation time, and a collimation beam pitch of 0.9. Pre-contrast images of the thoracic and cranial abdominal regions were obtained and reconstructed using a soft tissue algorithm in transverse, dorsal, and sagittal planes. Images were reviewed using a soft tissue window (WW 400, WL 60), a bone window (WW 1500, WL 300), and a lung window (WW 1600, WL-600). The contrast medium was administered intravenously into the cephalic vein via a 22-gauge catheter using a power injector (Leibel-Flarsheim 820740d injector system; Leibel-Flarsheim, Cincinnati, OH, USA) at an injection rate of 2 mL/second. A delayed phase scan was subsequently performed 2 min post-injection as part of the institution’s standard CT protocol. There was no evidence suggestive of mast cell tumor metastasis on the delayed phase images. On the 2 min delayed images, a generalized heterogeneous contrast enhancement of the hepatic parenchyma was observed, leading to a strong suspicion of hepatic congestion (Figure 1b). Marked edema of the esophageal wall, accompanied by intraluminal fluid retention, was observed alongside diffuse, intense mucosal hyperenhancement throughout the small intestinal tract (Figure 2b, Figure 3b and Figure 4b). The esophageal mural edema was most severe immediately cranial to the esophago-gastric junction (EGJ). 

To evaluate potential secondary changes, such as congestion within the right heart chambers and major vasculature, an additional delayed scan was performed at 4 min post-injection to further characterize the progressive CT changes and investigate the underlying hemodynamic mechanism. The anesthetist continuously monitored the patient’s ECG and arterial blood pressure through a viewing window that allowed direct observation of the patient. At the time of the 4 min delayed acquisition, no overt clinical signs of cardiovascular collapse were detected on the anesthetic monitor. The heart rate remained similar to the pre-contrast value at 80–100 beats/min, while the systolic arterial blood pressure was 120 mmHg, SpO_2_ was 100%, and EtCO_2_ was 35 mmHg. However, direct manipulation of the patient was avoided due to concerns regarding disruption of the established positioning. Consequently, direct physical examination, including assessment of body temperature, pulse, and mucous membranes, was limited during this interval. Given the absence of overt abnormalities in the monitored physiological parameters, the decision to obtain the additional delayed images was made at that time. 

On the 4 min delayed images, no findings such as right atrial enlargement or caudal vena cava (CVC) dilation, which could induce hepatic congestion, were observed. However, a decrease in esophageal wall thickening, an increase in intraluminal fluid retention within the esophagus, and gas-induced esophageal distension were noted (Figure 2c and Figure 3c). The esophageal wall thickness immediately cranial to the EGJ showed a transient increase at 2 min followed by a decrease at 4 min (Table 1). While the esophagus was entirely collapsed on pre-contrast scans, progressive distension filled with gas and fluid was observed from the thoracic inlet to the heart base starting from the 2 min delayed phase, with further aggravation over time.

**Figure 5:**
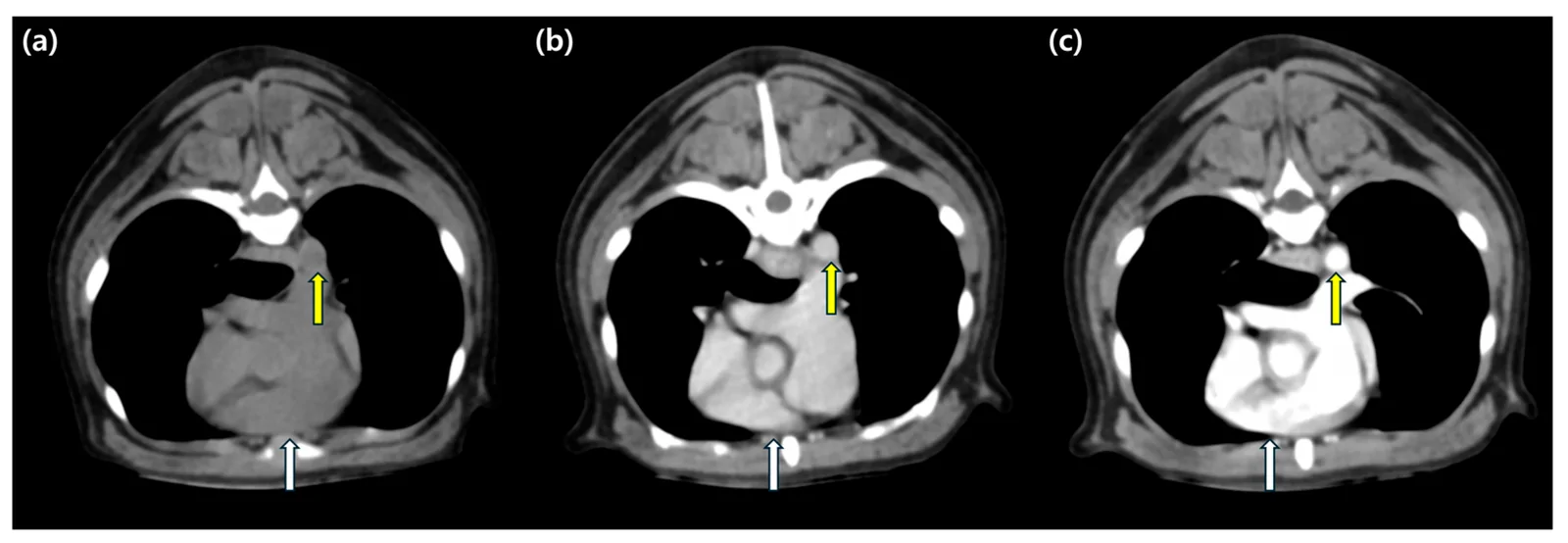
Changes in contrast enhancement within the heart (white arrows) and blood vessels, and alterations in the diameter of the descending aorta (yellow arrows). (**a**) Pre-contrast image. (**b**) 2 min delayed phase. Compared to the pre-contrast image, a mild decrease in the diameter of the descending aorta is observed. (**c**) 4 min delayed phase. No change in heart size is observed, but stagnation of the contrast medium within the heart is noted. The diameter of the descending aorta shows a mild decrease compared to the 2 min delayed phase.

Concurrently with the generalized vascular collapse depicted on the CT scans, contrast medium stagnation was observed within the major vasculature, including descending aorta, and CVC (Figure 2b,c and Figure 5b,c). Generalized vascular collapse was quantitatively assessed in the descending aorta at the level of the 6th thoracic vertebra, showing a sequential decrease in diameter across the CT phases (Table 1). Venous collapse was particularly prominent in the CVC and intra-abdominal veins. The short-axis diameter of the CVC was measured immediately cranial to the diaphragm and showed a marked progressive decrease across the pre-contrast, 2 min delayed, and 4 min delayed phases (Table 1). Distinct collapse of the hepatic veins was also noted. 

**Figure 6:**
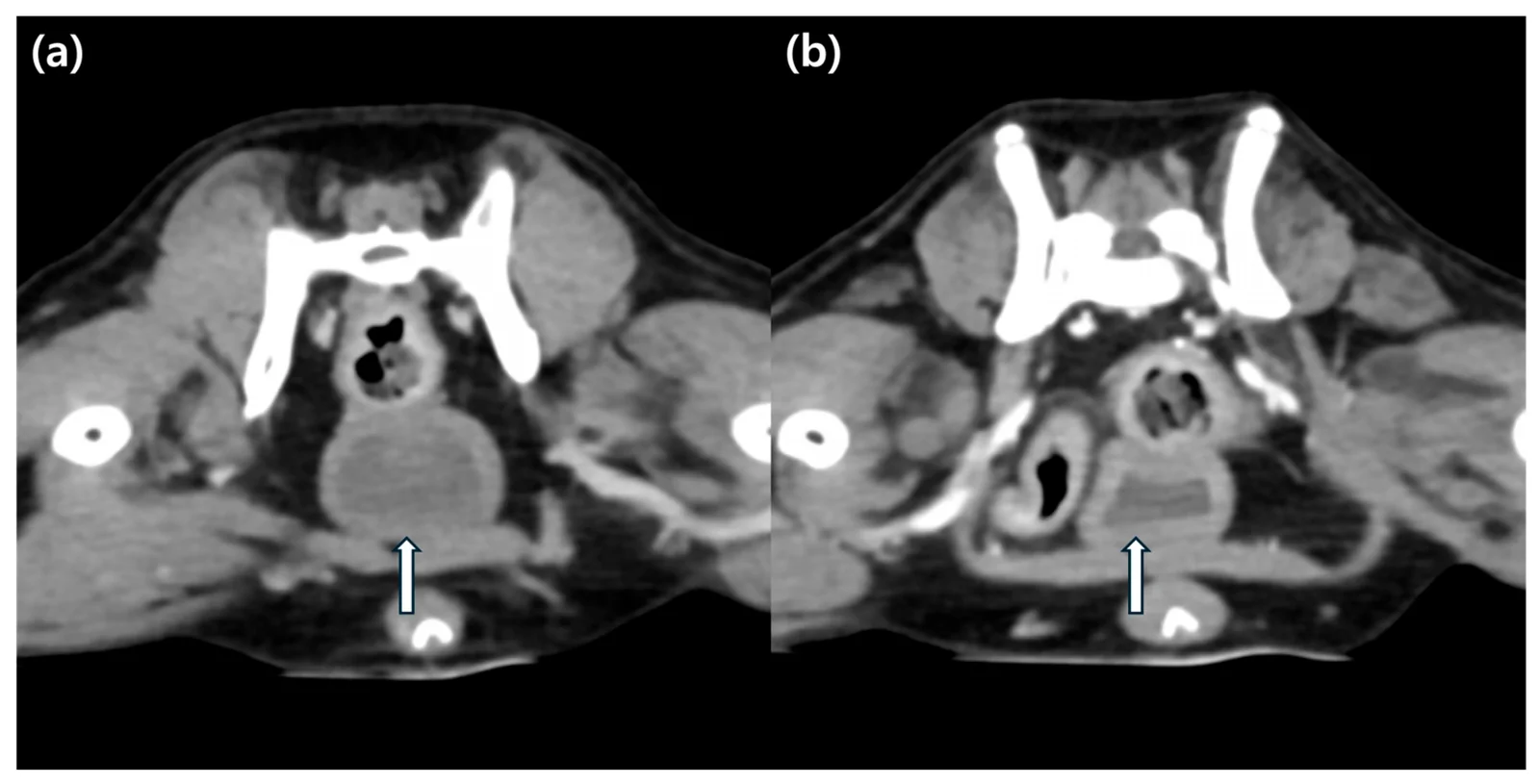
At the level of the sacrum, including the maximally distended urinary bladder (white arrows). (**a**) 2 min delayed phase. No accumulation of contrast medium is observed within the urinary bladder lumen. (**b**) 4 min delayed phase. Due to involuntary urination, a mild decrease in the size of the urinary bladder is observed, and no contrast medium is noted within the bladder. Concurrently, because there is no excretion of contrast medium from the kidneys, the contrast medium is not observed within the ureters and urinary bladder.

Stagnation of contrast medium was diffusely observed within the heart chambers and renal parenchyma, accompanied by the absence of the renal excretory phase. Contrast accumulation within the heart and kidneys was confirmed by an increase in HU values at 4 min compared to 2 min (Figure 4a–c and Figure 5b,c). Following the 2 min delay, contrast excretion into the renal pelvis, ureters, or urinary bladder was not observed (Figure 6a,b). This excretory failure was considered secondary to abrupt decline in glomerular filtration rate induced by profound systemic hypotension, leading to acute anuria [11]. Furthermore, contrast stagnation was widespread throughout the cephalic, thoracic, abdominal arterial and venous structures, with the exception of the portal vein, where no stagnation was observed. Intraluminal contrast attenuation within the arteries and veins was more pronounced at 4 min than at 2 min, with a progressive increase in mean attenuation within the CVC across the sequential CT phases (Table 1). This paradoxical increase in intravascular contrast attenuation was presumed to be caused by failure of renal contrast excretion, which consequently resulted in progressive stagnation and accumulation of the contrast medium within the major vasculature.

On the 4 min delayed scans, heterogeneous hepatic parenchymal enhancement remained similarly observable, whereas the intense mucosal hyperenhancement of the small intestines disappeared; however, an increase in intestinal corrugation and increased intraluminal gas were identified (Figure 1c and Figure 4c). Diffuse heterogeneous enhancement was consistently observed across all hepatic lobes on both 2 min and 4 min delayed phases. Strong mucosal enhancement of the small intestines peaked at 2 min and faded by 4 min. Intestinal corrugation was mild at 2 min but became prominent at 4 min, accompanied by a progressive increase in intraluminal gas over time. This gas accumulation was considered to be induced by ileus, which led to the loss of normal gastrointestinal motility and subsequent abnormal gas retention.

Following the 4 min delayed CT acquisition, during the anesthetic recovery period, the patient experienced a precipitous onset of respiratory failure and severe hypotension, with the systolic arterial blood pressure dropping below 50 mmHg, accompanied by cyanosis. Suspecting an acute anaphylactic reaction, emergency cardiopulmonary resuscitation (CPR) was initiated; with the endotracheal tube already in place, this included immediate 100 percent oxygen delivery and manual chest compressions. The patient was positioned in lateral recumbency, and chest compressions were performed for less than 1 min, after which return of spontaneous circulation (ROSC) was achieved. During CPR, accurate assessment of pulmonary compliance was difficult because chest compressions were performed concurrently with positive-pressure ventilation. Due to the urgent nature of the resuscitation field, the exact duration of the chest compressions and ventilatory support was not recorded. Following CPR, the ETCO_2_ gradually increased to >35 mmHg. Concurrently, a palpable pulse and measurable arterial pressure were restored, and these findings were considered consistent with ROSC. To manage the profound hypotension, an intravenous bolus of epinephrine (0.01 mg/kg) was promptly administered. Concurrently, as an emergency anti-inflammatory intervention against the suspected contrast-induced hypersensitivity, dexamethasone (0.2 mg/kg) was administered as an intravenous bolus. Subsequently, a continuous rate infusion (CRI) of dobutamine (5 mcg/kg/min, intravenously) was introduced to maintain a potent beta-1 mediated inotropic effect on the unstable cardiovascular system. 

**Figure 7:**
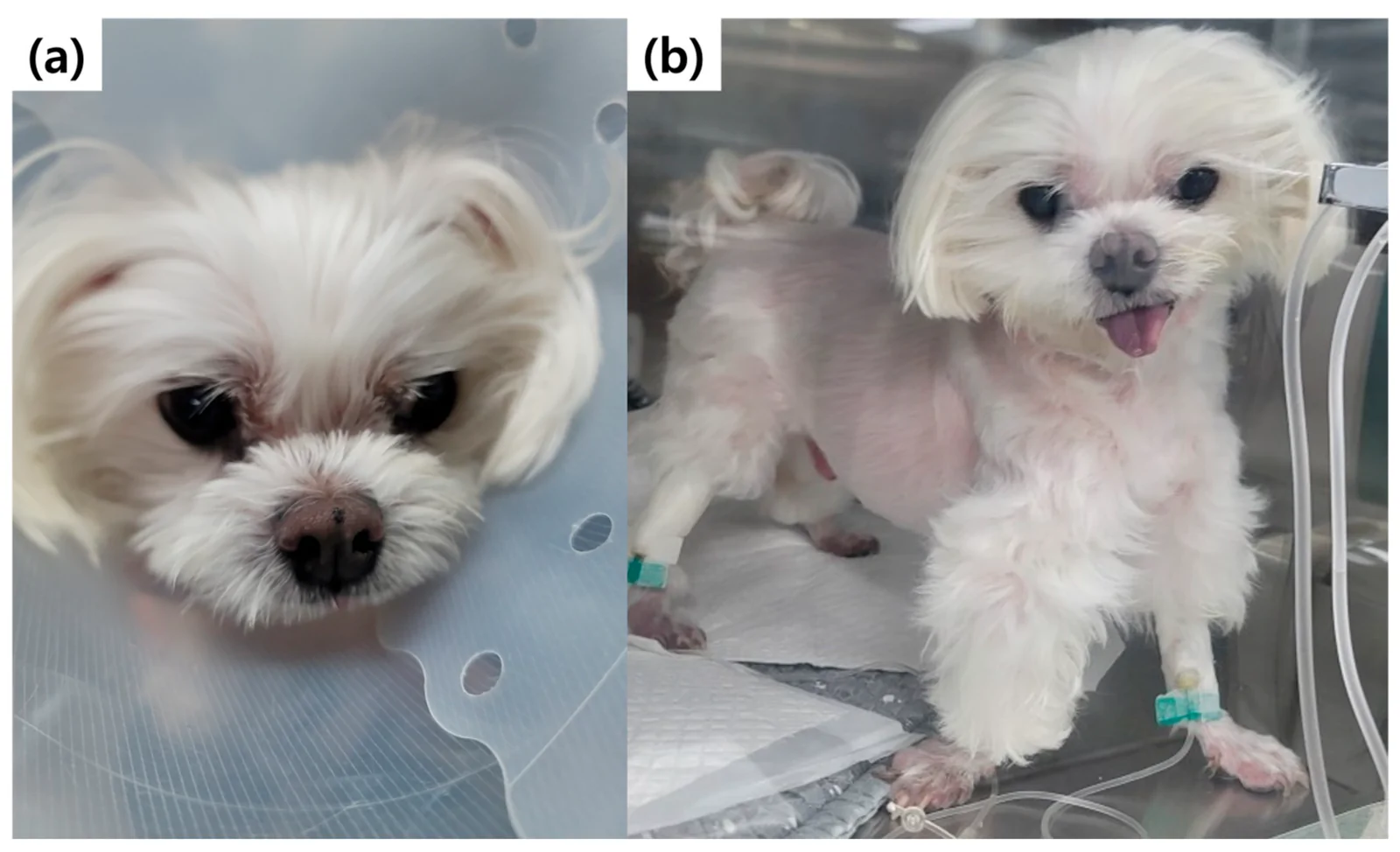
Gross appearance of the patient before (**a**) and after (**b**) the computed tomography (CT) scan. Bilateral periorbital edema and cyanosis are observed after the CT scan. Additionally, the patient exhibits ataxia and a wide-based stance due to an inability to support weight on the limbs.

During the post-resuscitation period, the patient’s systolic arterial blood pressure was closely and continuously monitored, with the dobutamine infusion rate adjusted accordingly. Immediately following resuscitation and during the early monitoring phase, clinical signs secondary to transient systemic hypoperfusion and hypersensitivity were observed, including periorbital edema, generalized ataxia, and transient cyanosis, all of which gradually resolved as systemic perfusion improved (Figure 7b). The patient regained an alert mental status approximately 2 h after CPR. Four hours post-initiation of dobutamine infusion, the systolic blood pressure stabilized at 150 mmHg, allowing the successful discontinuation of the CRI. However, during this subsequent monitoring phase, the patient developed transient hematochezia, followed by persistent watery diarrhea. 

At the owner’s request, the patient was discharged on the same day, which limited further short-term follow-up monitoring of hematological and renal functions within the hospital. However, serial telephonic follow-up was maintained with the owner. On the following day, the diarrhea resolved completely, and voluntary appetite returned to normal, although mild hindlimb ataxia persisted. Within a few days, all remaining neurological and gastrointestinal signs completely resolved, confirming a full, reversible recovery.

## 3. Discussion

In human medicine, hemodynamic alterations associated with contrast-induced anaphylactic shock have been extensively investigated [4,12,13,14,15,16,17]. Hypotension is the hallmark of an anaphylactic reaction, characterized by a precipitous drop in blood pressure due to rapid vasodilation and increased vascular permeability [12,14]. Although tachycardia typically develops as a compensatory mechanism against hypotension, bradycardia can concurrently occur due to a vagal response [4,5,8,10]. Contrast media reactions in veterinary medicine closely resemble those in humans, yet several species-specific manifestations have been documented. In dogs, clinical signs such as vomiting, generalized muscle twitching, facial edema, and diarrhea (including hematochezia) have been reported [10,18]. During severe reactions, profound hypotension often renders the femoral pulse weak or unpalpable [9,18]. Conversely, rare cases of concurrent severe hypertension and bradycardia have also been documented, potentially linked to a massive catecholamine release, autonomic nervous system responses, or underlying pathologies such as adrenal tumors [5,18]. Furthermore, bronchospasm during hypersensitivity reactions can lead to airway obstruction and ventilatory failure, which can be visualized as a narrowed airway on CT or indicated by a sudden drop in end-tidal carbon dioxide (EtCO2) concentrations [9,18]. In cats, vomiting, bradycardia, dyspnea, and (rarely) cardiac arrest may occur following contrast administration [2]. In the present case, the patient was considered to have experienced an acute, severe adverse reaction to a non-ionic contrast medium. Consistent with the previous human and veterinary literature, the patient exhibited hypotensive shock, respiratory arrest, and cardiac arrest. Following anesthetic recovery, cyanosis of the tongue, ataxia, periorbital edema, hematochezia, and persistent watery diarrhea were observed. Regarding the absence of a distinct drop in EtCO2 during the active CT imaging phase, it was primarily considered that bronchospasm might have developed with a temporal delay relative to other shock features, such as hypotension or esophageal edema, given that no definitive radiographic evidence of bronchospasm was observed on the CT scans at that time. Cutaneous signs, including flushing, pruritus, and urticaria, were not explicitly observed, which was likely due to these indicators being masked by the dense hair coat under general anesthesia. As the application of CT imaging continues to expand rapidly in veterinary medicine, radiologists and clinicians must meticulously monitor patients’ vital signs and clinical indicators—including end-tidal carbon dioxide (EtCO2), oxygen saturation (SpO2), electrocardiogram (ECG), systemic blood pressure, and potential cutaneous adverse reactions—both during and after contrast medium administration.

Mechanisms of adverse reactions to contrast media can be broadly classified into toxic reactions (Type A) and hypersensitivity reactions (Type B) based on their underlying causes [5,8,10]. Toxic reactions arise from the intrinsic physical properties of the contrast medium, such as osmolality, viscosity, and ionicity [7,8,17]. High-osmolality contrast media draw surrounding fluid into the vasculature, causing acute plasma volume expansion [5,10]. This can activate baroreceptors, leading to hypertension and bradycardia, or conversely, exacerbate hypotension through acute peripheral vasodilation [5,10,18]. Additionally, contrast molecules can exert a direct effect on the myocardium, inducing cardiac conduction system toxicity that reduces myocardial contractility, or damage vascular endothelial cells, thereby altering vascular reactivity [5,10]. On the other hand, hypersensitivity reactions occur independently of the dose administered and are further divided into anaphylactic and anaphylactoid reactions depending on the presence of immunological pathways [9,17]. The immunological (anaphylactic) mechanism is a classic allergic reaction mediated by immunoglobulin E (IgE) [9,12]. Although contrast reactions were historically considered predominantly non-immunological, recent studies have identified IgE-mediated allergy in more than 40% to 50% of cases [4,17]. Conversely, the non-immunological mechanism (anaphylactoid) involves the direct stimulation of mast cells and basophils by the contrast medium, triggering the release of chemical mediators such as histamine [9,12,17]. Furthermore, the complex activation of the complement and kinin systems, along with enzyme inhibition, cooperatively induces urticaria, bronchospasm, and vasodilation [8,9,17]. In particular, the rapid peripheral vasodilation and increased vascular permeability caused by histamine release lead to the extravasation of intravascular fluid into surrounding tissues, resulting in an abrupt drop in blood pressure and severe distributive shock [13,14,16]. In the present patient, the precipitous drop in blood pressure and widespread vessel collapse observed on CT strongly suggested a severe distributive shock driven by intravascular fluid extravasation, supporting a hypersensitivity-mediated volume depletion rather than a toxic reaction causing acute plasma volume expansion. Furthermore, given the patient’s medical history of oral mast cell tumor, we considered the possibility that this underlying condition might have lowered the threshold for mast cell activation and subsequent histamine release [12,17]. 

Contrast-induced nephropathy (CIN) involves a separate, multifactorial mechanism. The pathogenesis of CIN is primarily explained by a combination of renal hemodynamic changes and direct cytotoxicity [8]. Following an initial, transient increase in renal blood flow driven by the intrinsic chemotoxicity and high osmolality of the contrast medium, a compensatory reflex vasoconstriction ensues; this prolonged reduction in blood flow causes severe hypoxia, particularly within the renal medulla where oxygen tension is inherently low [8]. This is driven by potent vasoconstriction resulting from increased endothelin release and decreased nitric oxide synthesis [8]. Concurrently, direct contact of the contrast medium with renal tubular epithelial cells induces intracellular vacuolization and lysosomal alterations, while promoting the generation of reactive oxygen species to induce oxidative stress [5,8]. As these processes recur, the mitochondrial membrane potential of tubular cells is disrupted, ultimately culminating in apoptosis [8]. Clinical diagnosis of CIN is defined by an increase in serum creatinine levels of 25% or more, or 0.5 mg/dL or more, from baseline within 24 to 72 h post-exposure [5,7,8,18]. Although this patient did not manifest overt renal-related clinical signs such as oliguria or anuria following management, a key limitation remains that the co-occurrence of asymptomatic CIN could not be entirely ruled out due to the lack of post-CT serial evaluation of serum creatinine levels. Therefore, following contrast medium administration in procedures such as radiography, fluoroscopy, interventional radiology, and CT, veterinary radiologists are highly recommended to advise both the owners and referring clinicians regarding the potential development of CIN, thereby encouraging the subsequent monitoring of renal biomarkers.

In this patient, prominent CT abnormalities localized to the digestive tract served as crucial diagnostic indicators of severe contrast-induced anaphylactic shock. These features included progressive esophageal mural edema, gas- and fluid-induced esophageal distension, intense mucosal enhancement of the small intestine, and intestinal corrugation. Pathophysiologically, the rapid development of esophageal edema stems from immediate, histamine-mediated peripheral vasodilation and a dramatic surge in vascular permeability, which drives intravascular fluid into the interstitial space [14]. While a previously reported canine case of iohexol-induced Grade IV hypersensitivity demonstrated sustained, moderate esophageal wall thickening, the present patient exhibited a unique temporal change [9]. Specifically, the distinct esophageal wall thickening observed at 2 min post-injection paradoxically decreased on the 4 min delayed images. This rapid reduction in wall thickness over time is presumed to be secondary to the precipitous collapse of systemic systolic blood pressure below 50 mmHg. The ensuing severe visceral ischemia and hypoxia drastically deprived the esophageal vasculature of regional blood volume, causing the previously engorged wall to deflate and appear less thickened [19]. 

Concurrently, profound hypotension was presumed to activate the sympathetic nervous system, inducing intense vasoconstriction of the splanchnic vascular bed [8,19]. This ischemic stress paralyzes normal gastrointestinal motility—including swallowing and peristalsis—culminating in adynamic ileus and a progressively worsened intraluminal accumulation of fluid and gas [19]. This constellation of intraluminal fluid retention, wall thickening, and paradoxically intense mucosal enhancement under a shock state is collectively referred to as “Shock Bowel” [19]. The pathophysiological mechanism behind this mucosal hyperenhancement is directly attributed to the splanchnic vasoconstriction, which drastically slows capillary blood flow and traps the contrast medium within the mucosal vascular bed, preventing normal vascular washout and manifesting as marked hyperattenuation on CT [19]. When this prolonged ischemic insult is coupled with severe mucosal edema, it inevitably leads to the sloughing of the epithelial lining and microvascular hemorrhage, clinically manifesting as hemorrhagic enteritis with persistent watery diarrhea during the post-resuscitation period [14,18,19]. Therefore, when these distinct gastrointestinal hallmarks are identified on contrast-enhanced CT scans, radiologists must strongly suspect the development of the shock bowel complex and proactively advise clinicians to inform owners regarding the high probability of subsequent clinical complications during the recovery phase.

The patient demonstrated significant cardiovascular alterations on CT, characterized by marked collapse of the descending aorta and caudal vena cava (CVC), alongside diffuse intraluminal contrast media stagnation within the major vasculature, cardiac chambers, and renal parenchyma; this was concurrently associated with the absence of the renal excretory phase and signs of hepatic congestion. In both the human and veterinary imaging literature, flattening or a “slit-like” deformation of the vena cava has been recognized during severe anaphylactic shock [13,15]. Crucially, this feature can be captured on CT images prior to the overt manifestation of clinical shock symptoms, serving as an early surrogate marker for hemodynamic collapse [13]. During an anaphylactic cascade, a dramatic surge in vascular permeability allows intravascular fluid to rapidly extravasate into the interstitial space, with studies indicating a loss of up to 35% of the circulating plasma volume within 10 min [13,14,16]. The resulting relative hypovolemia significantly exacerbates the collapse of venous return, manifesting as severe narrowing of the CVC [14,15]. This systemic perfusion failure—driven by a precipitous decline in cardiac output and systemic hypotension—is clearly reflected by the delayed clearance and prolonged retention of contrast medium within both the cardiovascular and renal systems [5,8]. Specifically, the lack of sufficient hydrostatic driving pressure compromises the glomerular filtration rate (GFR), trapping the contrast agent within the renal tubules and causing functional anuria [8]. Simultaneously, the profound loss of forward vascular washout leads to mechanical stagnation of the contrast media within the cardiac chambers and great vessels [5,13].

The diffuse heterogeneous hepatic enhancement and congestion captured in this patient are presumed to be secondary to canine-specific shock organ dynamics, specifically driven by the acute constriction of the hepatic vein sphincters [20]. Unlike in humans, the canine hepatic veins possess well-developed smooth muscle sphincters that constrict potently in response to contrast-induced histamine release [20]. This targeted vasoconstriction induces acute hepatic venous outflow obstruction, leading to profound passive congestion and fluid pooling within the hepatic parenchyma [20]. 

In comparison with previously documented computed tomography (CT) features of contrast-induced adverse reactions and anaphylactic shock, the absence of several characteristic findings was noted in this patient. Notably, severe bronchospasm, compensatory splenic volume reduction, hyperenhancement of the adrenal medulla, and peripancreatic fluid attenuation (referred to as “Shock Pancreas”) were absent on the serial scans [9,18,19]. Regarding the absence of bronchospasm during the initial scans, although the patient developed overt respiratory distress during the post-contrast anesthetic recovery period, the exact timing of the onset of airway hyperreactivity could not be determined. One possible explanation is that airway hyperreactivity may have developed after completion of the 4 min delayed acquisition. Alternatively, the bronchodilatory effects of inhaled anesthetics, combined with mechanical endotracheal intubation, may have temporarily masked or suppressed clinical bronchospasm during the active scanning phase. [21]. However, in this patient, no obstructive pattern was observed during positive-pressure ventilation after CPR, suggesting that airway hypersensitivity was less likely to have occurred. Although the exact mechanism explaining why these specific signs were absent in this patient remains unknown, it would be highly valuable to evaluate whether these features are observed in other patients with suspected shock. Furthermore, future large-scale studies are warranted to investigate the exact incidence or frequency of these signs during the development of anaphylactic shock.

Severe adverse reactions occurring after contrast medium administration demand immediate and systematic management. Among these, the core management strategies common to both human and veterinary medicine include immediate discontinuation of the contrast agent, epinephrine administration, and airway stabilization [6,8,9,14,18]. Contrast administration must be halted as soon as signs of an adverse reaction are detected [8,18]. In human medicine, to maximize blood circulation, the patient is placed in a supine position with leg elevation; if vomiting occurs or consciousness is lost, the patient is placed in a lateral recumbent position to prevent aspiration [6]. Subsequently, as the most critical pharmacological intervention, the first-line drug epinephrine must be promptly administered [6,9]. In dogs, it is injected intravenously or intramuscularly at a dose of 0.005 to 0.01 mg/kg [2,9]. In cases of severe shock, a continuous rate infusion (CRI) may be more effective [22]. Although adjunctive medications such as antihistamines or corticosteroids can be considered, they should be administered primarily to alleviate cutaneous symptoms and prevent late-phase reactions; thus, epinephrine must take priority [6,8,22]. Glucagon is considered when patients taking beta-blockers do not respond to epinephrine, and atropine is administered when bradycardia (Bezold–Jarisch reflex) is concurrently present [2,6]. For respiratory and airway management, if there is a risk of airway obstruction due to laryngeal edema, rapid endotracheal intubation should be performed, and high-flow oxygen (6–10 L/min) must be supplied immediately [6,8]. If severe bronchospasm is present, an injectable beta-adrenoreceptor selective agonist (terbutaline, isoproterenol) or inhaled/nebulized salbutamol should be administered [6,23]. Following immediate management, continuous vital sign monitoring (ECG, SpO2, blood pressure) and intensive observation (at least 4 to 6 h, and over 24 h for severe cases) are required [6,13]. In this patient, the first-line drug epinephrine (0.01 mg/kg) was promptly administered intravenously, and dexamethasone (0.2 mg/kg) was administered anticipating its anti-inflammatory effects against the hypersensitivity reaction. Additionally, high-flow oxygen was immediately supplied under endotracheal intubation. However, the differences from the recommended guidelines were that the patient recovered normally without undergoing treatment for bronchospasm, and that a stable increase in blood pressure was successfully induced via a dobutamine CRI. Regarding dobutamine, there have been no previous reports of its use for managing shock occurring after contrast administration. In this patient, the application resulted in a stable blood pressure elevation without any adverse effects from dobutamine; nevertheless, large-scale studies regarding the safety of dobutamine following shock are considered necessary. As the use of contrast media gradually increases, it is crucial for radiologists to always consider the potential occurrence of such contrast medium-induced adverse reactions and to recognize the necessity of epinephrine application and immediate endotracheal intubation.

Several limitations exist within this case report. First, a multi-phase dynamic CT protocol including arterial, portal, and delayed sequences was not performed, which restricted a more time-resolved evaluation of regional perfusion alterations. Secondly, there was insufficient pre-emptive anticipation of clinical cardiovascular collapse prior to anesthetic recovery. At the time of the 2 min delayed acquisition, the patient’s anesthetic monitoring parameters remained within clinically acceptable ranges. Based on the clinical judgment at the time, an additional CT acquisition was therefore performed to further evaluate the cause and temporal progression of the esophageal mural edema and suspected hepatic congestion. However, this decision prolonged the duration of anesthesia, after which the patient developed an unexpected cardiovascular collapse. Therefore, the possibility that prolonged anesthesia may have contributed to or exacerbated the subsequent clinical deterioration cannot be completely excluded. This experience suggests that, even when anesthetic monitoring parameters remain stable, the CT findings described in this case should raise consideration of a possible hypersensitivity reaction, and the potential risks associated with further prolongation of anesthesia should be carefully considered. In such circumstances, early termination of anesthesia and initiation of appropriate clinical management should be considered rather than performing additional imaging solely to further characterize the evolving CT findings. Accordingly, veterinary imaging departments should maintain adequate clinical preparedness, and all personnel involved in contrast-enhanced imaging should be familiar with emergency management protocols for contrast-induced hypersensitivity reactions, even when low-osmolality non-ionic contrast media are used. Thirdly, a definitive rule-out of anesthesia-induced hypersensitivity remains impossible. Both anesthetic- and contrast-induced hypersensitivity reactions can be IgE-mediated, and both exhibit identical systemic manifestations ranging from cutaneous signs, such as urticaria and facial edema, to bronchospasm, precipitous hypotension, shock, and cardiac arrest; thus, differentiating between the two based solely on CT findings and clinical symptoms is inherently limited [9,23,24,25]. However, approximately 90% of anesthesia-induced hypersensitivity reactions in humans are reported to occur at the time of anesthetic induction, and cases in dogs also confirm the onset of symptoms immediately after endotracheal intubation [24,25]. Particularly, when propofol is used, the first 2–3 min post-injection are reported as the period of highest incidence [25]. In contrast, contrast-induced hypersensitivity reactions predominantly occur within 5 min immediately following contrast medium administration [5,13,17,18]. Therefore, considering this temporal difference, the patient showed no abnormalities 2–3 min after anesthetic induction, and the CT alterations were observed immediately following contrast injection; thus, a contrast-induced hypersensitivity reaction was primarily presumed.

## 4. Conclusions

This case provides a uncommon report of acute severe hypersensitivity induced by a non-ionic contrast medium in a canine patient, with real-time systemic alterations captured sequentially via CT. Based on the clinical manifestations, the patient exhibited profound hypotension, respiratory failure, facial edema, hematochezia, and diarrhea, which correlated with hallmark CT features including severe vascular collapse, diffuse contrast stagnation within the heart and renal parenchyma, hepatic congestion, marked esophageal wall edema, and intense intestinal mucosal enhancement. Despite the life-threatening status, the immediate initiation of a comprehensive resuscitation protocol—comprising cardiopulmonary resuscitation, rapid epinephrine administration, and a sequential dobutamine CRI—successfully reversed the cardiovascular failure, restoring the patient to normal clinical condition. Ultimately, this report underscores that veterinary radiologists and supporting technical staff must remain thoroughly educated on the potential occurrence of these acute contrast-induced adverse events during imaging procedures, emphasizing the necessity of maintaining immediate preparedness to initiate prompt, systematic emergency interventions.

## Figures and Tables

**Table 1:** Sequential quantitative changes in esophageal wall thickness, vascular dimensions, and contrast attenuation across CT phases.

Phase	Esophageal Wall Thickness ^1^ (mm)	Descending Aorta Diameter ^2^ (mm)	CVC Diameter ^3^ (mm)	Aorta Attenuation ^4^ (HU)	CVC Attenuation ^5^ (HU)
Pre-contrast	3.2	7.5	9.4	8.6	2.4
2 min delayed	10.1	6	7.5	184.3	134
4 min delayed	4.7	5	5.3	268.9	301

^1^ Esophageal wall thickness was measured immediately cranial to the EGJ, where the most prominent changes were observed. ^2^ The descending aorta was measured at the level of the 6th thoracic vertebra, where the most prominent changes in diameter were observed, with the vessel wall included in the measurement. ^3^ The CVC was measured immediately cranial to the diaphragm, where the most prominent changes in diameter were observed, including the vessel wall in the measurement. ^4^ Average of the mean HU values measured in the lumens of three different cross-sections of the descending aorta. ^5^ Average of the mean HU values measured in the lumens of three different cross-sections of the CVC.

## Data Availability

The data presented in this study are available in the article.
